# Effect of storage time and the level of formic acid on fermentation characteristics, epiphytic microflora, carbohydrate components and *in vitro* digestibility of rice straw silage

**DOI:** 10.5713/ajas.20.0388

**Published:** 2021-04-22

**Authors:** Jie Zhao, Siran Wang, Zhihao Dong, Junfeng Li, Yushan Jia, Tao Shao

**Affiliations:** 1 Institute of Ensiling and Processing of Grass, College of Agro-grassland Science, Nanjing Agricultural University, Nanjing 210095, China; 2 Key Laboratory of Forage Cultivation, Processing and High Efficient Utilization of Ministry of Agriculture, Inner Mongolia Agricultural University, Hohhot 010018, China

**Keywords:** Formic Acid, Fermentation Type, Epiphytic Microflora, Carbohydrate Components, *In vitro* Fermentation, Rice Straw Silage

## Abstract

**Objective:**

The study aimed to evaluate the effect of storage time and formic acid (FA) on fermentation characteristics, epiphytic microflora, carbohydrate components and *in vitro* digestibility of rice straw silage.

**Methods:**

Fresh rice straw was ensiled with four levels of FA (0%, 0.2%, 0.4%, and 0.6% of fresh weight) for 3, 6, 9, 15, 30, and 60 d. At each time point, the silos were opened and sampled for chemical and microbial analyses. Meanwhile, the fresh and 60-d ensiled rice straw were further subjected to *in vitro* analyses.

**Results:**

The results showed that 0.2% and 0.6% FA both produced well-preserved silages with low pH value and undetected butyric acid, whereas it was converse for 0.4% FA. The populations of enterobacteria, yeasts, moulds and aerobic bacteria were suppressed by 0.2% and 0.6% FA, resulting in lower dry matter loss, ammonia nitrogen and ethanol content (p<0.05). The increase of FA linearly (p<0.001) decreased neutral detergent fibre and hemicellulose, linearly (p<0.001) increased residual water soluble carbohydrate, glucose, fructose and xylose. The *in vitro* gas production of rice straw was decreased by ensilage but the initial gas production rate was increased, and further improved by FA application (p<0.05). No obvious difference of FA application on *in vitro* digestibility of dry matter, neutral detergent fibre, and acid detergent fibre was observed (p>0.05).

**Conclusion:**

The 0.2% FA application level promoted lactic acid fermentation while 0.6% FA restricted all microbial fermentation of rice straw silages. Rice straw ensiled with 0.2% FA or 0.6% FA improved its nutrient preservation without affecting digestion, with the 0.6% FA level best.

## INTRODUCTION

As of today, in Southeast Asia as well as southwest China, farmers still use rice straw as the main or even sole roughage source for their livestock. Although fibre-like materials are necessary for stimulating rumination, direct feeding of straw to ruminants fails to meet their nutritional needs due to the rough texture, high fibre proportion and low degraded nutrients of the straw [1]. Meanwhile, the seasonal generation of straw requires suitable preservation methods to provide a year-round feed supply. Faced with the realities of the humid and hot climate in these regions, the conventional ensilage technology has been widely applied as a preferred method for feed preservation compared to the drying process.

It cannot be neglected that most straw is ensiled with high fermentation losses even if the fermentation accelerant is added. In our previous study [2], high ammonia nitrogen (NH_3_-N) and ethanol content were produced not only in control but also in rice straw ensiled with molasses or enzyme and lactic acid bacteria (LAB). Considering the relatively low protein and water soluble carbohydrates (WSC) content in rice straw, it seems particularly important to reduce protein and substrate losses during ensiling. Therefore, fermentation inhibitors such as formic acid (FA) could be a potential additive for straw silage production to reduce nutrients loss. For animal production, restrictively fermented silage may result in higher energy yield of rumen microbes than the extensively fermented silage, because more soluble sugars remained in restrictively fermented silage [3]. It is known that soluble sugars are a better source of energy for rumen microbes than fermentative acids [4].

Interestingly, there are inconsistent results in previous studies about the effects of FA on fermentation of silage. Researchers found that FA application inhibited [5,6], did not affect [3], or even aided [7,8] lactic fermentation of silages. For instance, Chamberlain and Quig [4] reported that FA applied at 6 mL/kg level inhibited silage fermentation of perennial ryegrass silage but aided the silage fermentation at 2 mL/kg level. While He et al [9] concluded that 2 mL/kg of FA suppressed the fermentation of *Neolamarckia cadamba* leaves silage. These results indicated that silage fermentation might be either promoted or inhibited by FA application depending on the application level, material type, and other factors. However, to our knowledge, there are no available data about the effect of FA application level and storage time on the fermentation of rice straw silage.

Thus, the study evaluated the effect of four levels of FA on 60-d fermentation dynamics, epiphytic microflora, carbohydrate components and *in vitro* digestibility of rice straw silage, and the optimal application level of FA was also determined.

## MATERIALS AND METHODS

### Animal care

The experimental protocol was approved by the Animal Care and Use Committee and performed under the institutional guidelines for animal experiments of the College of Animal Science and Technology of Nanjing Agriculture University and the recommendations proposed by the European Commission (1997) to minimise the suffering of animals.

### Materials and silage preparation

Rice straw was collected after grain harvest from the field of Nanjing Branch of Chinese National Centre for Rice Improvement in Jiangsu Academy of Agricultural Science (Jiangsu, China), leaving the stubble of 20 cm. The harvested rice straw is yellow-green with hollow and tough stems. Then the rice straw was cut into about 2 cm by a fodder chopper and 0.3 kg straw was collected by the method of quartering for the analyses of pre-ensiled properties, chemical and microbial compositions before ensiling. The chopped straw was treated with 0% (control), 0.2%, 0.4%, and 0.6% fresh weight (FW) of FA (purity, 98%; Sinopharm Chemical Reagent Co., Ltd., Shanghai, China). Specifically, 20 mL/kg FW additives or distilled water was sprayed into the corresponding samples. After thoroughly mixing, approximately 520 g treated material was tightly packed into a laboratory silo (1 L polyethylene bottle with a height of 18.7 cm and diameter of 9.5 cm) and sealed by two screw tops and plastic tape. All silos were stored at the ambient temperature (22°C to 28°C), and five silos per treatment were opened for the following analyses on 3, 6, 9, 15, 30, and 60 d after ensiling, respectively.

### Chemical and microbial analyses

After initial buffering capacity (BC) analysis [10], the fresh or ensiled rice straw was devided into three subsamples. The first subsample was homogenized with distilled water at 1:3 w/v ratio and stored at 4°C for 24 h. The extract was filtered by two layers of gauze and a filter paper for later determination of pH, NH_3_-N, organic acids and ethanol. The pH of fresh or ensiled rice straw was recorded with a glass electrode pH meter. After 10,000×g centrifugation for 10 min, the supernatant fluid was analysed for NH_3_-N [11] as well as organic acids and ethanol [2].

The second subsample was blended well with sterilized saline solution (0.85% NaCl) at 1:9 w/v ratio and then used for 6-fold serial dilution. The population of LAB, aerobic bacteria (AB) and yeasts and moulds were enumerated on de Man, Rogosa and Sharp agar medium, nutrient agar medium and potato dextrose agar medium (Sincere Biochemical Technology Co., Ltd., Shanghai, China) after incubation at 37°C for 3 d, respectively. Moreover, enterobacteria were enumerated on Violet Red Bile Glucose agar medium after incubation at 37°C for 1 d.

The remaining subsample was immediately freeze-dried to determine the dry matter (DM) content. And then the dried sample was ground to pass through a 1-mm screen for analyses of crude protein (CP), structural carbohydrates, non-structural carbohydrates and crude ash (Ash). Total nitrogen was measured by a Kjeldahl N analyser (Kjeltec 8200; Foss Analytics, Höganäs, Sweden) and multiplied by 6.25 to convert to CP. Neutral detergent fibre (NDF) and acid detergent fibre (ADF) were analysed following the procedures of Van Soest et al [12] by an Ankom 200 Fibre Analyzer (Ankom Technology, Macedon, NY, USA). The WSC was determined via the colorimetric method [13]. The monosaccharides including glucose, xylose and fructose were determined according to the method of Desta et al [14]. Ash was measured by incinerating in a muffle furnace at 550°C for 4 h.

### Evaluation of silage quality

To evaluate the silage quality, the V-score (an evaluation method that calculated from volatile fatty acids and NH_3_-N) was adopted [15]. The silage quality can be evaluated by the 100-point scale as below: 81 to 100 (good), 60 to 80 (moderate), and <60 (bad).

### *In vitro* incubation of fresh and 60-d ensiled rice straw

The rumen fluid collected from two rumen-fistulated Holstein cows (about 100 kg body weight) before morning feeding was immediately mixed, filtered and stored at 39°C in a water bath for incubation. The cows freely accessed drinking water and were fed twice daily with the diet based on corn silage at 1.2 times the maintenance level. Prior to use, the inoculum was prepared by mixing the rumen fluid with an artificial saliva solution at a 1:2 (v/v) ratio [16]. The preparation process was conducted under continuous CO_2_ flushing to keep the anaerobic condition.

The *in vitro* incubation was performed in serum bottles as described by Zhao et al [2]. Briefly, 1 g ground sample was weighed into an F57 filter bag (Ankom Technology, USA). The filter bag was previously washed with acetone, dried at 65°C for 24 h and weighed. All bags were well-sealed and placed into the corresponding 120 mL serum bottles. And another six serum bottles without bag added were as blank. Then, 60 mL inoculum was added into each bottle under CO_2_ flushing at 39°C. The volume of gas production (GP) was read after 4, 8, 12, 24, 36, 48, and 72 h of incubation by a calibrated syringe and corrected with blank.

The data of GP were fitted to an exponential model of Blümmel et al [17]: y = b (1−e^−ct^), in which y is the cumulative volume of GP at incubation time t (mL), b is the potential GP (mL) and c is the rate constant of GP.

After 72-h incubation, the filter bags were gently rinsed with distilled water until clean and oven-dried at 65°C for 48 h to constant weight. *In vitro* DM digestibility (IVDMD) and NDF digestibility (IVNDFD), and ADF digestibility (IVADFD) were determined based on their respective weight differences before and after incubation.

### Statistical analysis

Microbial data were transformed to the logarithmic form on an FW basis. Data on fermentation characteristics, epiphytic microflora and carbohydrate components were subjected to two-way analysis of variance (ANOVA) with the fixed effects of FA levels, ensiling days and their interaction, and data on CP, Ash and *in vitro* parameters were subjected to one-way ANOVA using the general linear model procedure of SAS (Version 9.1, SAS Institute, Cary, NC, USA). Polynomial contrasts (linear and quadratic) were performed to examine the effects of the equally spaced FA level. Tukey’s multiple comparisons were used to determine the statistical difference between means, and the level of significance was set at p<0.05. Pearson correlation heatmap was used to clarify the relationship among fermentation characteristics, epiphytic microflora and carbohydrate components in this study using R software (Version 2.15.3).

## RESULTS AND DISCUSSION

### Characteristics of raw rice straw

For ensiling, the available sugar and epiphytic LAB in the material are the critical factors to determine the quality of silage. As shown in Table 1, the DM, WSC (>5% DM) and FC value (>35) of the rice straw fulfil the theoretical requirement for well-fermented silage [18]. However, the population of epiphytic LAB was less than the minimum level of 5.0 log_10_ cfu/g FW for satisfactory fermentation [19]. Meanwhile, the high counts of enterobacteria (8.56 log_10_ cfu/g FW) and AB (6.27 log_10_ cfu/g FW) also posed a challenge to produce high-quality rice straw silage. Considering that the pH of the rice straw can be easily lowered due to its low BC (41.6 mEq/kg DM), the combining ensilage with acid seems to give a way out for the rapid acidification and effective preservation of the straw material.

### Fermentation dynamics of rice straw silage

**Figure 1:**
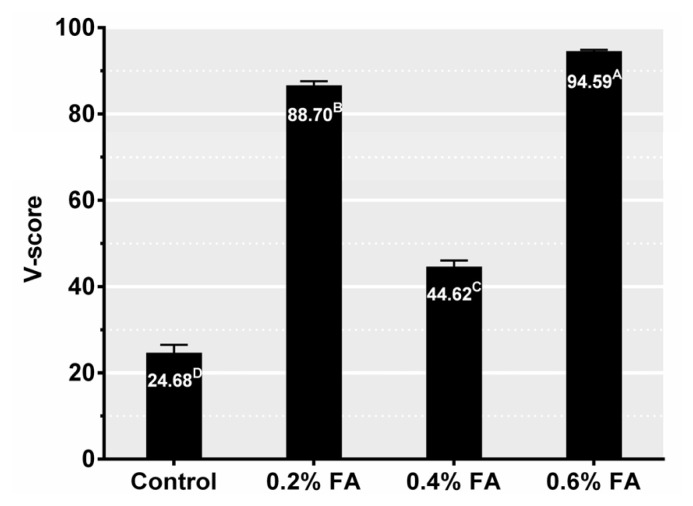
V-score of 60-d rice straw silage. Treatments: control, no additive; 0.2% FA, 0.2% formic acid; 0.4% FA, 0.4% formic acid; 0.6% FA, 0.6% formic acid (n = 5, bars indicate standard error of the means). Means with different letters in the column (^A–D^) are significant at p<0.05.

The fermentation parameters of rice straw silages are illustrated in Table 2. Ensiling time, FA application level and their interaction significantly influenced the pH and organic acids content (p<0.05). The pH in ensiled rice straw decreased linearly (p<0.001) by increasing levels of FA application and this could be responsible for the direct acidification from FA. Unexpectedly, the pH of 0.4% FA treatment increased (p<0.05) rather than decreased at the late stage of ensiling, which is consistent with the finding of Leibeinsperger and Pitt [20] that the final pH of silages can either be increased or decreased depending on the FA application level. The high final pH lead to the silage quality of 0.4% FA being worse than that of 0.2% FA and 0.6% FA, evidenced by the high butyric acid (BA) content and low V-score (Figure 1). The unexpected observation that the initial rapid acidification of 0.4% FA could not induce successful fermentation is difficult to interpret but could be attributed that the intermediate level of FA inhibiting LAB but not inhibiting undesirable microbes, which is reflected in the low LA content and high BA content. It is reported that FA has less inhibitory effect on enterobacteria as this type of microbe can produce FA [18].

After an initial inhibition, the increase of lactic acid (LA) in 0.2% FA was higher than that in control, and the highest (p<0.05) LA content was recorded in 0.2% FA after 30 d of ensiling, indicating that 0.2% FA aided the LA fermentation of rice straw silages. Also, the high LA/acetic acid found in 0.2% FA was indicative of a predominantly homo-fermentation. The results about the positive effect of a low level of FA (0.2% FA) on silage fermentation have also been observed in previous studies [8,21]. Different from the other treatments, the pH value and fermentation acids of 0.6% FA silage always remained at a low or undetectable level throughout the entire ensiling process, which is a typical inhibitory performance of FA applied at a high level [5]. Namely, 0.6% FA severely restricted the fermentation of rice straw silages by the direct acidification and antimicrobial properties. From the above, both low and high levels of FA produced well-preserved rice straw silages, but in different ways.

### Epiphytic microflora of rice straw silage

The characteristics of the silage depend on the microflora of material being ensiled and on the conditions that promote or inhibit the microflora [22]. Ensiling time, FA application level and their interaction significantly affected the microbial populations of rice straw silage (Table 3, p<0.01). The population of LAB in all treatments showed a downward trend after the initial rise, and this could be explained that WSCs were depleted at the late stage of ensiling or the final pH fell outside the optimal range [23]. Although 0.2% FA significantly (p<0.05) suppressed the proliferation of LAB in the early period of ensiling, these organisms eventually attained a high population at the end of ensiling, even higher (p<0.05) than that of natural fermentation (control). One possible reason is that FA has an aliphatic structure and might be utilized as an energy source for LAB under low concentration [24]. The populations of LAB and mould were decreased (p<0.05) at 0.4% FA level while enterobacteria and yeasts were not affected, suggesting that FA at this level was effective in preventing the growth of LAB and moulds but not enterobacteria and yeasts. Indeed, enterobacteria and yeasts have been found to be tolerant of FA [18]. The appearance of moulds at the late stage of ensiling in control and 0.4% FA was associated with the increase of pH [22], and the gradual absence of AB as ensilage proceed was related to the depletion of oxygen.

### Fermentation losses of rice straw silage

As shown in Table 4, the effects of ensiling time and FA application level were significant on the DM content and fermentation losses of rice straw silages (p<0.001). With the increase of FA application level, the DM content in ensiled rice straw increased curvilinearly, while the NH_3_-N and ethanol content decreased curvilinearly (p<0.05). The DM loss, NH_3_-N and ethanol content respectively reflect the extent of total fermentation loss, protein degradation and energy waste during ensiling.

Overall, the FA application effectively inhibited the NH _3_-N and ethanol production in the present study, which is consistent with Yuan et al [5]. However, it is worth noting that high DM loss, NH_3_-N and ethanol content were produced in 0.4% FA subsequently even with an initial inhibition. This result is surprising but corresponds well with the increase of pH in 0.4% FA silage. Similarly, high fermentation losses were generated in other materials ensiled with 0.4% FA [4,25]. Enterobacteria and yeasts are found to be tolerant to FA [18]. It is inferred that 0.4% FA was not sufficient to inhibit the proliferation of these microbes, resulting in large quantities of NH_3_-N and ethanol production in 0.4% FA at the late stage of ensiling. As for 0.2% FA, it is the rapid proliferation of LAB as aforementioned instead of FA that inhibits the activities of undesired microbes and reduces the fermentation losses, which is in line with Chamberlain and Quig [4]. While the deactivation of plant proteinase and broad antimicrobial activities induced by 0.6% FA was responsible for the low DM loss, NH_3_-N and ethanol content in 0.6% FA.

### Structural carbohydrate composition of rice straw silage

The NDF and hemicellulose content of rice straw silages was affected (p<0.05) by ensiling time and FA application level, and the cellulose content was affected (p<0.05) by ensiling time (Table 5). It is well known that the degradation of structural carbohydrates during ensiling can be contributed by the action of enzymes, acids and microbes. In this study, acidolysis as the main factor could account for the alteration of structural carbohydrate components. The FA application, especially at the 0.6% FA level, significantly (p<0.05) decreased the NDF and hemicellulose content, while only a numerical (p>0.05) decrease was found on ADF and cellulose as expected. Several studies have shown that FA can selectively degrade the hemicellulose component but has only a minor effect on the cellulose component of biomass [26,27]. Similarly, Ren et al [6] found a larger NDF reduction than of ADF in FA-treated silages. These results suggested that hemicellulose is more susceptible to degradation than cellulose during ensiling, which is corresponds with the previous studies [2,18]. Furthermore, the hemicellulose content in 0.6% FA was still lower (p<0.05) than that in the original straw even with fermentation loss, indicating areal decrease of hemicellulose. The higher ADF, cellulose and acid detergent lignin content in silages versus raw material could be due to the fermentation losses resulted from the non-fibrous component. The lignin component is difficult to degrade under conventional ensiling conditions.

### Nonstructural carbohydrate composition of rice straw silage

**Figure 2:**
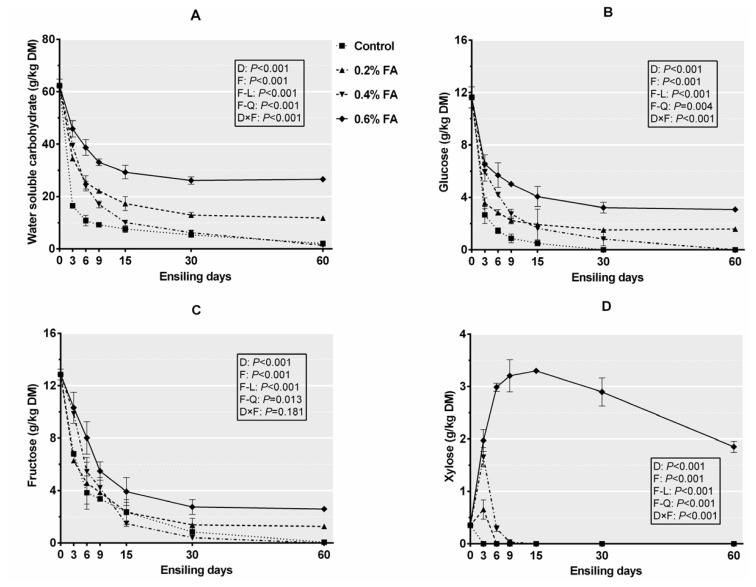
(A) Water soluble carbohydrates, (B) glucose, (C) fructose, and (D) xylose of rice straw silage. DM, dry matter. Treatments: control, no additive; 0.2% FA, 0.2% formic acid; 0.4% FA, 0.4% formic acid; 0.6% FA, 0.6% formic acid (n = 5, bars indicate standard error of the means). D, ensiling time; F, formic acid application level; F-L and F-Q are linear and quadratic effects of application level, respectively; D×F, interaction of ensiling time and application level.

Figure 2 exhibits the dynamic changes of nonstructural carbohydrate components of rice straw silage. Ensiling time, FA application level and their interaction affected (p<0.05) the content of WSC, glucose and xylose, and the WSC, fructose and xylose content in ensiled rice straw increased linearly and quadratically with the increase of FA application level (p< 0.05). As ensilage proceeded, all nonstructural carbohydrates except xylose showed a clear downtrend, but the reduction of 0.2% FA and 0.6% FA was slower (p<0.05) than that of control and 0.4% FA, suggesting that 0.2% and 0.6% FA reduced additional sugar consumption and preserved more nonstructural carbohydrate contents. This could ascribe to the inhibition of substrate depletion from plant respiration and microbial activities under low pH conditions. The presence of a larger xylose content in 0.6% FA (Figure 2D) indicated that the hemicellulose fraction of rice straw silages was effectively hydrolysed to xylose at the high FA level, which was well reflected by the compositional variation of hemicellulose (Table 5). Xylose as a typical pentose is derived from hemicellulose degradation [2]. Meanwhile, the small amounts of xylose detected in 0.2% FA and 0.4% FA at d 3 of ensiling could also be attributed to acidolysis of hemicellulose resulted from the initial rapid acidification. The larger (p<0.05) decline of glucose and fructose than that of xylose indicated that hexose could be the more favoured substrate for silage microbes than pentose. Deng et al [28] also demonstrated that hexose (such as glucose) is easier to be utilized as compared to pentose (such as xylose).

### Correlation coefficient analysis

**Figure 3:**
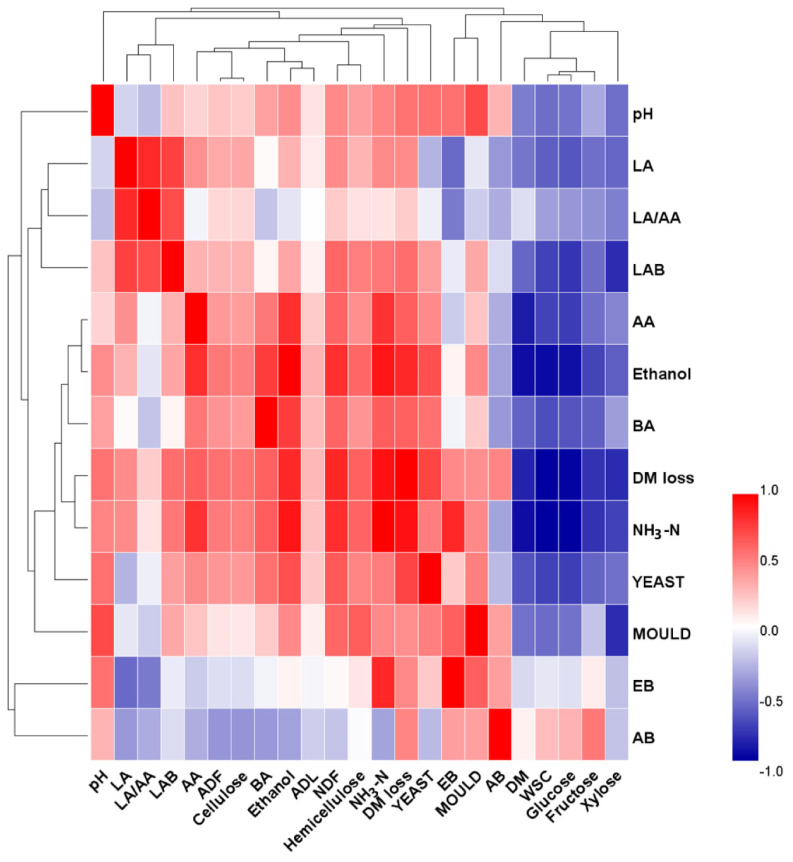
Pearson’s correlation heatmap of fermentation characteristics, epiphytic microflora and carbohydrate components of rice straw silages. Red squares represent positive correlation, whereas blue squares represent negative correlation. DM, dry matter; LA, lactic acid; AA, acetic acid; LA/AA, ratio of lactic acid to acetic acid; BA, butyric acid; LAB, lactic acid bacteria; EB, enterobacteria; AB, aerobic bacteria; NH_3_-N, ammonia nitrogen; NDF, neutral detergent fibre; ADF, acid detergent fibre; ADL, acid detergent lignin; WSC, water soluble carbohydrates.

Pearson’s correlation heatmap (Figure 3) was constructed to elucidate the relationships among fermentation characteristics, epiphytic microflora and carbohydrate components. The correlation analysis showed that NDF (r = 0.497, p<0.001) and hemicellulose (r = 0.417, p<0.001) were positively related to pH value. It could be considered that hemicellulose is the easily degradable fraction of structural carbohydrates [2], which was effectively degraded by the application of FA. The negative correlation of WSC (r = −0.480, p<0.001), glucose (r = −0.463, p<0.001), fructose (r = −0.260, p<0.05) and xylose (r = −0.478, p<0.001) with pH value suggested that low pH obtained higher soluble sugars by the inhibition of sugar consumption and the degradation of structural carbohydrates. In addition to clostridia, NH_3_-N is also produced by enterobacteria (r = 0.853, p<0.001), accompanied by high BA (r = 0.658, p<0.001) and ethanol (r = 0.920, p<0.001) content. The increase of DM loss is associated with high pH (r = 0.576, p< 0.001), BA (r = 0.645, p<0.001), ethanol (r = 0.852, p<0.001) and NH_3_-N (r = 0.933, p<0.001) content, which is mainly due to the proliferation of yeasts (r = 0.748, p<0.001) and moulds (r = 0.544, p<0.001). The strong correlation between ethanol and yeasts (r = 0.728, p<0.001) confirmed the fact that ethanol is mainly produced by yeasts during ensiling [18].

### Crude protein, ash, and *in vitro* parameters of 60-d rice straw silage

The higher (p<0.05) CP content in 0.2% FA and 0.6% FA relative to control and 0.4% FA could attributed to the low pH in the corresponding silages suppressing the proteolysis induced by plant enzyme and proteolytic bacteria. The difference in Ash content could be associated with DM loss since Ash was expressed on a DM basis (Table 6).

**Figure 4:**
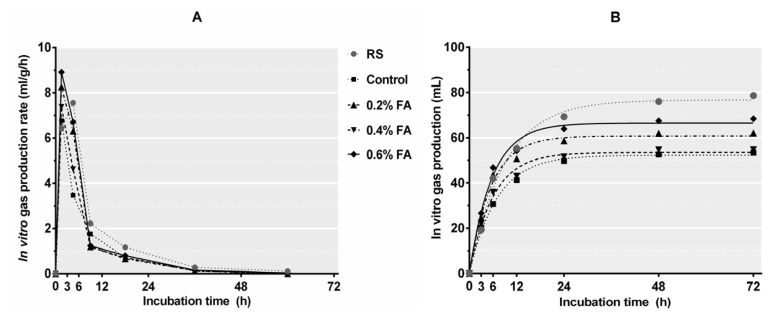
Gas production rate (A, mL/g/h) and profiles (B, mL/g dry matter) from *in vitro* fermentation of rice straw silages for 72 h. Treatments: RS, fresh rice straw; control, no additive; 0.2% FA, 0.2% formic acid; 0.4% FA, 0.4% formic acid; 0.6% FA, 0.6% formic acid (n = 5).

*In vitro* GP and digestibility has gained wide acceptance as a means to evaluate the nutritional value of ruminant feeds. Meanwhile, the relationship between these two parameters is commonly used to predict the actual DM intake and feeds digestion of ruminants [29]. In this study, the initial GP rate (3 h of incubation) was increased (p<0.05) after ensilage, and further improved (p<0.05) by 0.2% FA or 0.6% FA (Figure 4A), demonstrating a faster adaptation of rumen microorganisms to these silages. Meanwhile, the *in vitro* GP of rice straw was decreased (p<0.05) after ensilage (Figure 4B), which is consistent with Zhao et al [2]. This result could be related to the fermentation loss. All FA application levels, especially 0.2% FA and 0.6% FA, increased b value and *in vitro* GP of the resulting silages (Table 6; Figure 4). Similar result was also found by Zhang et al [30] that FA increased *in vitro* GP of wild ryegrass silages and this could be explained by the higher WSC content in FA-treated silages. While, the FA application only numerically affected IVDMD, IVNDFD, and IVADFD, which is consistant with the study of Aksu et al [3] that FA application from 0.2% to 0.6% had no substantial impact on the ruminal utilization of rice straw silages.

## CONCLUSION

In the present study, the 0.2% FA application level promoted LA fermentation while 0.6% FA restricted all microbial fermentation of rice straw silages. Both 0.2% and 0.6% FA inhibited undesirable microbes, reduced fermentation loss, enhanced hemicellulose degradation and maintained more residual sugars thereby producing well-preserved rice straw silages. Whereas the potential risk observed in 0.4% FA level should be noted because this level inhibited the activity of LAB but not enterobacteria and yeasts. Application of FA at 0.6% performed best on nutrient preservation without affecting digestibility and can be recommended for the production of rice straw silage with low fermentation losses.

## Figures and Tables

**Table 1:** Chemical composition, pre-ensiled properties and microbial population of rice straw before ensiling

Items	Rice straw
Chemical composition (% DM)	
DM (% FW)	42.4
CP	6.14
WSC	6.23
Glucose	1.16
Fructose	1.28
Xylose	0.04
NDF	60.3
ADF	37.1
ADL	5.05
Cellulose	32.0
Hemicellulose	23.2
Pre-ensiled properties	
pH	6.43
BC (mEq/kg DM)	41.6
FC	54.4
Microbial population (log_10_ cfu/g FW)	
LAB	4.51
EB	8.56
Yeasts	5.20
Moulds	4.56
AB	6.27

DM, dry matter; FW, fresh weight; CP, crude protein; WSC, water soluble carbohydrate; NDF, neutral detergent fibre; ADF, acid detergent fibre; ADL, acid detergent lignin; BC, buffering capacity; mEq, milligram equivalent; FC, fermentation coefficient; cfu, colony-forming units; LAB, lactic acid bacteria; EB, enterobacteria; AB, aerobic bacteria.

**Table 2:** Effect of ensiling time and formic acid application level on fermentation parameters during ensiling

Items	Treatments1)	Ensiling days						Mean	SEM	p-value2)				
	
		3	6	9	15	30	60			D	F	F-L	F-Q	D×F
pH	Control	5.44^Aa^	5.23^Aab^	4.65^b^	4.72^ABb^	4.68^ABb^	4.78^ABb^	4.92^A^	0.052	0.007	<0.001	<0.001	0.085	<0.001
	0.2% FA	5.06^Ba^	5.03^Aa^	4.49^b^	4.30^Cb^	4.19^Bb^	4.28^Bb^	4.56^B^						
	0.4% FA	4.32^Cc^	4.64^Bbc^	4.52^bc^	4.80^Aabc^	5.18^Aab^	5.43^Aa^	4.82^A^						
	0.6% FA	4.03^Ca^	4.24^Ca^	4.13^a^	4.33^BCa^	4.28^ABa^	4.30^Ba^	4.27^C^						
Lactic acid (% DM)	Control	1.23^Ac^	1.92^Ac^	2.51^Aabc^	3.67^Aab^	3.92^Aa^	2.28^Bbc^	2.59^A^	0.210	<0.001	<0.001	<0.001	0.020	<0.001
	0.2% FA	0.49^Bc^	1.18^Bbc^	1.81^ABbc^	3.49^Aab^	5.78^Aa^	5.54^Aa^	3.05^A^						
	0.4% FA	0.05^Bc^	0.11^Cc^	0.27^Bbc^	0.63^Bab^	0.81^Bab^	1.14^Ca^	0.50^B^						
	0.6% FA	0.04^Bc^	0.07^Cc^	0.16^Bbc^	0.11^Bbc^	0.42^Bab^	0.71^Ca^	0.25^B^						
Acetic acid (% DM)	Control	0.69^Ae^	0.83^Ade^	1.01^Ad^	1.30^Ac^	2.24^Ab^	2.58^Aa^	1.44^A^	0.072	<0.001	<0.001	<0.001	<0.001	<0.001
	0.2% FA	0.30^Bc^	0.38^Bbc^	0.40^Babc^	0.52^Babc^	0.60^Bab^	0.63^Ba^	0.47^B^						
	0.4% FA	0.23^Bd^	0.19^Cd^	0.25^Ccd^	0.37^Bbc^	0.46^BCb^	0.74^Ba^	0.37^C^						
	0.6% FA	0.23^Bc^	0.20^Cc^	0.20^Cc^	0.25^Bbc^	0.44^Ca^	0.38^Cab^	0.28^D^						
Butyric acid (% DM)	Control	ND^b^	ND^b^	ND^b^	0.04^b^	0.17^Aa^	0.21^Aa^	0.07^A^	0.010	<0.001	<0.001	<0.001	0.028	<0.001
	0.2% FA	ND	ND	ND	ND	ND^B^	ND^B^	0.00^B^						
	0.4% FA	ND^b^	ND^b^	0.03^b^	0.04^b^	0.22^Aa^	0.27^Aa^	0.09^A^						
	0.6% FA	ND	ND	ND	ND	ND^B^	ND^B^	0.00^B^						
LA/AA	Control	1.77^Aab^	2.31^Aab^	2.46^ABa^	2.84^ABa^	1.75^Bab^	0.88^Cc^	2.00^B^	0.032	<0.001	<0.001	<0.001	<0.001	<0.001
	0.2% FA	1.62^Ac^	3.32^Abc^	4.43^Aabc^	7.44^Aabc^	9.57^Aa^	8.80^Aab^	5.86^A^						
	0.4% FA	0.20^B^	0.57^B^	1.08^B^	2.01^AB^	1.72^B^	1.55^BC^	1.19^BC^						
	0.6% FA	0.17^Bb^	0.38^Bb^	0.80^Bb^	0.45^Bb^	1.02^Bab^	1.90^Ba^	0.78^C^						

SEM, standard error of means; DM, dry matter; LA/AA, ratio of lactic acid to acetic acid; ND, not detected.

1) Control, no additive; 0.2% FA, 0.2% formic acid; 0.4% FA, 0.4% formic acid; 0.6% FA, 0.6% formic acid;

2) D, ensiling time; F, formic acid application level; F-L and F-Q are linear and quadratic effects of application level, respectively; D×F, interaction of ensiling time and application level.

Means (n = 5) with different letters in the same row (^a–e^) or column (^A–D^) are significant at p<0.05.

**Table 3:** Effect of ensiling time and formic acid application level on the microbial population during ensiling

Items (log_10_ cfu/g FW)	Treatments1)	Ensiling days						Mean	SEM	p-value2)				
	
		3	6	9	15	30	60			D	F	F-L	F-Q	D×F
LAB	Control	5.64^Aabc^	6.23^Aa^	5.91^Aab^	5.53^Bbc^	5.20^Bcd^	4.75^Bd^	5.54^B^	0.158	<0.001	<0.001	<0.001	<0.001	<0.001
	0.2% FA	4.15^Bd^	5.68^Bc^	6.09^Abc^	6.72^Aa^	7.05^Aa^	6.13^Ab^	5.97^A^						
	0.4% FA	3.38^Cc^	3.43^Cc^	3.69^Bbc^	4.25^Cb^	5.10^Ba^	4.93^Ba^	4.13^C^						
	0.6% FA	2.53^Dd^	2.74^Dcd^	3.13^Bbc^	3.33^Db^	3.98^Ca^	2.90^Cbcd^	3.10^D^						
EB	Control	8.43^Aa^	7.70^Aab^	7.11^Ab^	6.86^Ab^	3.89^Cc^	3.94^Cc^	6.32^A^	0.255	<0.001	<0.001	<0.001	0.375	<0.001
	0.2% FA	7.92^Aa^	7.24^Ab^	6.81^Ac^	6.59^Ac^	ND^Dd^	ND^Dd^	4.76^B^						
	0.4% FA	5.95^Bb^	6.10^Bab^	6.35^Bab^	6.71^Aab^	6.99^Aa^	6.53^Aab^	6.44^A^						
	0.6% FA	3.61^Cc^	3.83^Cbc^	3.99^Cb^	5.62^Ba^	5.69^Ba^	5.88^Ba^	4.77^B^						
Yeasts	Control	5.25^Aa^	4.86^Aab^	4.82^Aab^	4.75^Aab^	4.23^Ab^	4.59^Aab^	4.75^A^	0.063	<0.001	<0.001	<0.001	0.468	0.001
	0.2% FA	3.94^Acd^	4.52^Aab^	4.76^Aa^	4.02^Abcd^	4.38^Babc^	3.81^Bd^	4.24^BC^						
	0.4% FA	3.89^Bc^	3.91^Bc^	3.94^Bc^	4.56^Abc^	5.39^Aa^	5.07^Aab^	4.46^AB^						
	0.6% FA	3.78^Bb^	3.82^Bb^	3.82^Bb^	4.09^Aab^	4.23^Ba^	3.91^Bb^	3.94^C^						
Moulds	Control	4.92^A^	4.80^A^	4.31^A^	3.73^A^	3.38^A^	3.45^A^	4.10^A^	0.211	<0.001	<0.001	<0.001	<0.001	<0.001
	0.2% FA	4.05^Ba^	4.12^ABa^	3.89^Aa^	2.56^Bb^	ND^Bc^	ND^Bc^	2.44^C^						
	0.4% FA	2.48^Cc^	3.55^Bab^	4.06^Aab^	4.32^Aa^	4.42^Aa^	3.29^Abc^	3.69^B^						
	0.6% FA	ND^D^	ND^C^	ND^B^	ND^C^	ND^B^	ND^B^	0.00^D^						
AB	Control	7.03^Aa^	6.41^Ab^	ND^Bc^	ND^c^	ND^c^	ND^c^	2.24^A^	0.269	<0.001	<0.001	<0.001	<0.001	<0.001
	0.2% FA	4.57^Cb^	5.02^Ba^	ND^Bc^	ND^c^	ND^c^	ND^c^	1.60^B^						
	0.4% FA	5.31^BCa^	4.50^Cb^	4.64^Ab^	ND^c^	ND^c^	ND^c^	2.41^A^						
	0.6% FA	3.68^Da^	ND^Db^	ND^Bb^	ND^b^	ND^b^	ND^b^	0.61^C^						

cfu, colony-forming units; FW, fresh weight; SEM, standard error of means; LAB, lactic acid bacteria; EB, enterobacteria; ND, not detected; AB, aerobic bacteria.

1) Control, no additive; 0.2% FA, 0.2% formic acid; 0.4% FA, 0.4% formic acid; 0.6% FA, 0.6% formic acid.

2) D, ensiling time; F, formic acid application level; F-L and F-Q are linear and quadratic effects of application level, respectively; D×F, interaction of ensiling time and application level.

Means (n = 5) with different letters in the same row (^a–d^) or column (^A–D^) are significant at p<0.05.

**Table 4:** Effect of ensiling time and formic acid application level on DM and fermentation losses of rice straw silages

Items	Treatments1)	Ensiling days						Mean	SEM	p-value2)				
	
		3	6	9	15	30	60			D	F	F-L	F-Q	D×F
DM (% FW)	Control	35.2^Ba^	32.7^Bbc^	34.2^Bab^	32.8^Bbc^	32.0^Bbc^	31.8^Cc^	33.1^C^	0.380	<0.001	<0.001	<0.001	<0.001	0.613
	0.2% FA	40.0^A^	39.3^A^	38.9^A^	38.1^A^	37.7^A^	36.8^A^	38.5^B^						
	0.4% FA	39.5^Aab^	40.2^Aa^	38.6^Aabc^	36.9^ABabc^	35.7^ABbc^	34.5^Bc^	37.6^B^						
	0.6% FA	40.7^A^	40.6^A^	40.8^A^	41.0^A^	39.2^A^	38.6^A^	40.1^A^						
DM loss (% DM)	Control	8.90^Ac^	12.8^Ab^	12.4^Abc^	14.3^Aab^	15.4^Aab^	16.8^Aa^	13.4^A^	0.559	<0.001	<0.001	<0.001	0.015	0.018
	0.2% FA	6.70^Ab^	8.84^ABb^	10.0^ABab^	10.7^Aa^	10.8^ABa^	11.3^Ba^	9.72^B^						
	0.4% FA	3.69^Bd^	6.74^ABcd^	8.73^ABcd^	11.3^Abc^	15.7^Aab^	17.1^Aa^	10.5^B^						
	0.6% FA	2.81^Bc^	3.23^Bab^	3.34^Bab^	4.20^Bab^	5.47^Bab^	6.76^Ca^	4.30^C^						
NH_3_-N (% TN)	Control	9.89^Ac^	13.0^Abc^	13.8^Abc^	14.9^Ab^	16.4^Aab^	19.8^Aa^	14.6^A^	0.546	<0.001	<0.001	<0.001	0.011	<0.001
	0.2% FA	5.43^Bb^	6.68^Bb^	7.57^Bab^	8.78^ABa^	9.07^Ca^	9.87^Ba^	8.57^B^						
	0.4% FA	3.36^Bd^	5.76^Bcd^	7.44^Bbc^	9.96^ABb^	13.9^Ba^	18.9^Aa^	9.89^B^						
	0.6% FA	3.18^Bc^	3.97^Bc^	4.12^Cbc^	5.12^Babc^	6.39^Dab^	7.03^Ca^	4.97^C^						
Ethanol (% DM)	Control	0.82^Ad^	1.23^Ac^	1.39^Abc^	1.63^Ab^	2.04^Aa^	2.14^Aa^	1.54^A^	0.065	<0.001	<0.001	<0.001	<0.001	<0.001
	0.2% FA	0.42^Bb^	0.62^Bab^	0.70^BCab^	0.77^Ca^	0.73^Ca^	0.61^Bab^	0.64^C^						
	0.4% FA	0.38^BCd^	0.60^Bcd^	0.82^Bbc^	1.09^Bb^	1.59^Ba^	1.76^Aa^	1.04^B^						
	0.6% FA	0.32^Cd^	0.39^Bcd^	0.44^Cbc^	0.52^Cab^	0.59^Ca^	0.54^Bab^	0.47^D^						

SEM, standard error of means; DM, dry matter; FW, fresh weight; NH_3_-N, ammonia nitrogen; TN, total nitrogen.

1) Control, no additive; 0.2% FA, 0.2% formic acid; 0.4% FA, 0.4% formic acid; 0.6% FA, 0.6% formic acid.

2) D, ensiling time; F, formic acid application level; F-L and F-Q are linear and quadratic effects of application level, respectively; D×F, interaction of ensiling time and application level.

Means (n = 5) with different letters in the same row (^a–d^) or column (^A–D^) are significant at p<0.05.

**Table 5:** Effect of ensiling time and formic acid application level on structural carbohydrate composition of rice straw silages

Items	Treatments1)	Ensiling days						Mean	SEM	p-value2)				
	
		3	6	9	15	30	60			D	F	F-L	F-Q	D×F
NDF (% DM)	Control	63.2^Ad^	66.1^Ac^	68.0^Ab^	68.3^Ab^	69.2^Ab^	71.1^Aa^	67.7^A^	0.496	<0.001	<0.001	<0.001	<0.001	<0.001
	0.2% FA	62.0^ABb^	64.1^Bab^	66.1^ABa^	66.0^ABa^	65.6^Ba^	64.9^Bab^	64.8^B^						
	0.4% FA	61.0^Cd^	63.3^Bcd^	64.9^ABbcd^	66.5^Aabc^	68.5^Aab^	71.3^Aa^	65.9^AB^						
	0.6% FA	59.8^Ca^	58.6^Cab^	58.4^Cab^	58.7^Bab^	58.4^Cab^	57.9^Cb^	58.6^C^						
ADF (% DM)	Control	39.0^c^	40.4^bc^	41.5^ab^	41.9^ab^	42.0^ab^	42.9^a^	41.3	0.292	0.005	0.058	0.010	0.694	0.268
	0.2% FA	38.2	39.1	40.0	40.7	41.0	41.1	40.0						
	0.4% FA	37.5^d^	38.9^cd^	39.3^bc^	40.0^bc^	42.0^ab^	43.4^a^	40.2						
	0.6% FA	38.0	38.3	38.9	39.6	39.6	39.8	39.0						
ADL (% DM)	Control	5.33	5.47	5.42	5.55	5.58	5.87	5.54	0.061	0.544	0.423	0.250	0.649	0.916
	0.2% FA	5.22	5.35	5.23	5.31	5.29	5.35	5.29						
	0.4% FA	5.15	5.27	5.29	5.39	5.57	5.97	5.44						
	0.6% FA	5.11	5.18	5.22	5.33	5.29	5.36	5.25						
Cellulose (% DM)	Control	33.7^b^	34.9^ab^	36.1^a^	36.3^a^	36.5^a^	37.0^a^	35.8	0.265	0.007	0.072	0.013	0.727	0.131
	0.2% FA	33.0^b^	33.7^ab^	34.8^ab^	35.4^a^	35.7^a^	35.7^a^	34.7						
	0.4% FA	32.4^b^	33.6^ab^	34.0^ab^	34.7^ab^	36.4^a^	37.5^a^	34.7						
	0.6% FA	32.9	33.1	33.7	34.3	34.3	34.4	33.8						
Hemicellulose (% DM)	Control	24.2^Ac^	25.7^Abc^	26.5^Aab^	26.4^Aab^	27.2^Aab^	28.2^Aa^	26.4^A^	0.399	0.730	<0.001	<0.001	<0.001	0.139
	0.2% FA	23.8^A^	25.1^A^	26.1^A^	25.3^AB^	24.7^B^	23.8^B^	24.8^B^						
	0.4% FA	23.5^Ac^	24.4^Abc^	25.6^Aab^	26.4^Aab^	26.5^Aab^	27.9^Aa^	25.7^A^						
	0.6% FA	21.9^Ba^	20.3^Bab^	19.5^Bab^	19.0^Bab^	18.8^Cab^	18.1^Cb^	19.6^C^						

SEM, standard error of means; NDF, neutral detergent fibre; DM, dry matter; ADF, acid detergent fibre; ADL, acid detergent lignin.

1) Control, no additive; 0.2% FA, 0.2% formic acid; 0.4% FA, 0.4% formic acid; 0.6% FA, 0.6% formic acid.

2) D, ensiling time; F, formic acid application level; F-L and F-Q are linear and quadratic effects of application level, respectively; D×F, interaction of ensiling time and application level.

Means (n = 5) with different letters in the same row (^a–d^) or column (^A–C^) are significant at p<0.05.

**Table 6:** Crude protein, Ash, gas production kinetics and *in vitro* degradability of fresh rice straw and 60-d rice straw silages

Items	Treatments1)				SEM	p-value2)		
	
	Control	0.2% FA	0.4% FA	0.6% FA		F	F-L	F-Q
CP (% DM)	5.89^B^	6.27^A^	6.05^AB^	6.35^A^	0.044	0.009	0.009	0.617
Ash (% DM)	14.9^A^	13.3^B^	15.0^A^	12.8^B^	0.318	0.001	0.007	0.335
Potential gas production, b (mL)	52.3^C^	60.8^B^	53.5^C^	66.5^A^	1.473	<0.001	<0.001	0.034
Gas production rate constant, c (mL/h)	0.15	0.18	0.17	0.18	0.012	0.052	0.035	0.195
*In vitro* dry matter degradability (%)	53.5	52.5	51.0	53.6	1.221	0.279	0.801	0.106
*In vitro* neutral detergent fibre degradability (%)	51.4	49.5	50.7	49.0	2.430	0.852	0.755	0.787
*In vitro* acid detergent fibre degradability (%)	44.5	45.3	44.8	45.6	2.838	0.725	0.418	0.701

SEM, standard error of means; CP, crude protein; DM, dry matter; Ash, crude ash.

1) Control, no additive; 0.2% FA, 0.2% formic acid; 0.4% FA, 0.4% formic acid; 0.6% FA, 0.6% formic acid.

2) F, formic acid application level; F-L and F-Q are linear and quadratic effects of application level, respectively.

Means (n= 5) with different letters in the same row (^A–C^) are significant at p<0.05.
